# Development of a Synthetic VP1 Protein Peptide-Based ELISA to Detect Antibodies Against Porcine Bocavirus Group 3

**DOI:** 10.3390/v16121946

**Published:** 2024-12-19

**Authors:** Chao Gong, Hui He, Yuguang Fu, Baoyu Li, Bin Yang, Jianlong Li, Xiaodong He, Juncheng Han, Yi Zhang, Guangliang Liu, Qingyong Guo

**Affiliations:** 1 Xinjiang Key Laboratory of New Drug Study and Creation for Herbivorous Animals (XJ-KLNDSCHA), College of Veterinary Medicine, Xinjiang Agricultural University, Urumqi 830052, China; 18328042911@163.com (C.G.); 15109020563@163.com (H.H.); pbb0108@163.com (J.L.); shouyihexiaodong@sina.com (X.H.); hanjuncheng6@163.com (J.H.); zydy052@163.com (Y.Z.); 2 State Key Laboratory for Animal Disease Control and Prevention, College of Veterinary Medicine, Lanzhou Veterinary Research Institute, Chinese Academy of Agricultural Sciences, Lanzhou University, Lanzhou 730000, China; fuyuguang@caas.cn (Y.F.); libaoyu@caas.cn (B.L.); yangbin02@caas.cn (B.Y.)

**Keywords:** peptide-based ELISA, VP1, porcine bocavirus group 3

## Abstract

Porcine bocavirus (PBoV), classified within the genus Bocaparvovirus, has been reported worldwide. PBoV has been divided into group 1, group 2, and group 3. PBoV group 3 (G3) viruses are the most prevalent in China. Currently, effective serological methods for the detection of antibodies against PBoV G3 are limited. In this study, we developed an indirect ELISA using a synthetic VP1 peptide designed on the basis of the conserved region of the PBoV VP1 protein as a coating antigen. Through matrix titration, the optimal coating concentration of the VP1 peptide (0.5 μg/mL), serum dilution (1:200), and working concentration of the secondary antibody (1:50,000) were determined. The cutoff value of this developed ELISA was set as 0.4239. Further investigations revealed that this developed ELISA had no cross-reactivity with positive serum antibodies against FMDV-O, FMDV-A, PRV, ASFV, SF, PCV2, PEDV, and TGEV. The detection limit of the method was a 1:1600 dilution of standard positive serum against PBoV G3. The coefficients of variation for both the intra- and interassay data were lower than 10%. A total of 1373 serum samples collected from 12 provinces in China between 2022 and 2023 were subjected to indirect ELISA. The results showed that 47.56% of the samples were PBoV G3 positive. These results reveal that peptide-based ELISA is a reliable and cost-effective method for detecting PBoV G3 antibodies. It also facilitates the investigation of the prevalence and distribution of PBoV G3.

## 1. Introduction

Parvoviruses, which belong to the Parvoviridae family, are responsible for a wide range of diseases in animals [1]. The Parvoviridae family is further divided into two subfamilies: Densovirinae and Parvovirinae. The genus Bocaparvovirus within the Parvovirinae subfamily encompasses various viruses, including human bocavirus (HBoV), porcine bocavirus (PBoV), canine minute virus (CMV), bovine bocavirus (BPV), gorilla bocavirus (GBoV), and California sea lion bocavirus (CslBoV) [2,3]. PBoV was first identified in Sweden in 2009 from swine lymph nodes afflicted with postweaning multisystemic wasting syndrome (PMWS) [4]. Subsequently, PBoV was reported in Europe, Asia, North America, and Africa [5,6,7]. PBoV can be detected in various types of tissue, including lymph nodes [4], lungs, saliva [8], intestines [9], and spleen [10], suggesting that PBoV may invade these tissues and that it may be associated with diseases [7].

PBoV is a nonenveloped virus with a linear single-stranded DNA genome of approximately 5–6 kb [11]. The genome comprises three primary open reading frames (ORFs) designated ORF1, ORF2, and ORF3. ORF1 encodes nonstructural protein 1 (NS1); ORF2 encodes viral capsid proteins 1 and 2 (VP1/2), with VP1 encompassing the entire VP2 sequence along with an additional N-terminal region [5]; and ORF3 encodes nuclear protein 1 (NP1). PBoV is categorized into different clades on the basis of the VP1 and VP2 nucleotide sequences, including PBoV1, PBoV2, PBoV3, PBoV4, PBoV5, PBoV3C, PBoV-6 V, and PBoV-7 V. However, PBoV can be classified into three distinct groups, PBoV G1, PBoV G2, and PBoV G3, according to the sequence of the complete VP1 gene [5,12,13]. Due to the involvement of bocavirus in respiratory tract infection as well as gastrointestinal tract infection, it is still unclear whether bocaviruses are respiratory pathogens or enteric pathogens, especially in humans, pigs, and other animals. Due to the lack of an experimental animal model and cell-line-adapted PBoV strain, the pathogenic nature of PBoV has not been investigated [14]. The pathogenesis of PBoV has not been determined to date. It is thought that, as PBoV is highly prevalent in the swine population and has high genetic diversity, its pathogenesis could be determined by direct evidence from clinical disease [7]. Reports suggest that some PBoVs may be associated with respiratory signs or diarrhea, although the pathogenicity of PBoVs has also not been clearly recognized, mainly due to a lack of a suitable cell culture system or animal model [4,15].

Previous studies have shown that the rate of PBoV positivity has increased in China [6,16,17] and that PBoV G3 is more prevalent compared with PBoV G1 and PBoV G2 [13]. Currently, immortalized cell lines for the isolation and propagation of PBoV are not available, and basic research is difficult. In addition, PBoV has been not only detected in pigs suffering from PMWS but also detected in pigs suffering diarrhea [16,18,19], poor growth [20], respiratory illness [21], and encephalomyelitis [22]. Therefore, developing powerful methods to investigate the distribution and prevalence of PBoV, which facilitates the prevention and control of PBoV, is essential. Immunological methods, such as indirect immunofluorescence assays, have been developed to detect unidentified isolated PBoV in primary porcine kidney cell lines [23]. The ELISA method is suitable for large-scale detection of serum samples, and the procedure is relatively simple and less time-consuming. Several ELISA methods have been reported for the detection of antibodies against PBoV [16,23,24]. However, because PBoV shows a high degree of genetic diversity, there are limited ELISA methods available for detecting PBoV G3 antibodies.

The VP1 structural protein encoded by ORF2 is the main antigenic protein of PBoV and largely reflects the antigenicity of the virus [25], suggesting that this protein is a potential antigen for the detection of antibodies against PBoV. In this study, a conserved amino acid peptide of the VP1 protein was synthesized as a coating antigen to develop an ELISA method for the detection of PBoV G3 antibodies. The cross-reactivity, sensitivity, reproducibility, and coefficients of variation of this ELISA were evaluated. This ELISA was also used to analyze 1373 field serum samples. The ELISA developed in this study provides a reliable tool for the detection of PBoV G3 and facilitates the prevalence survey of PBoV G3 in China.

## 2. Materials and Methods

### 2.1. Preparation of the VP1 Protein Peptide

Based on the amino acid (aa) sequence of all available PBoV G3 VP1 proteins in the GenBank database, the conserved peptides were analyzed. The similarity of the peptides of PBoV G3 VP1 proteins were compared with the PBoV G1 and G2 VP1 proteins in the GenBank database via the MegAlign program of DNAStar software (version 7.1, DNASTAR Inc., Madison, WI, USA). The antigenicity of the peptides was analyzed via the Protean program (Version 15.1.0 (155); DNASTAR Inc., Madison, WI, USA), and the primary peptides were subjected to GL Biochem (Shanghai, China) Ltd. for synthesis.

### 2.2. Preparation of Standard Negative and Positive Porcine Sera Against PBoV G3

Prior to acquiring a one-week-old piglet, we conducted health screenings using RT-qPCR to detect foot-and-mouth disease virus O subtype (FMDV-O), foot-and-mouth disease virus A subtype (FMDV-A), porcine circovirus 2 (PCV2), swine fever virus (SF), African swine fever virus (ASFV), pseudorabies virus (PRV), porcine epidemic diarrhea virus (PEDV), transmissible gastroenteritis virus (TGEV), and PBoV. A one-week-old clinically healthy pig was orally administered 2 mL (approximately 8.9 × 106 genome copies per milliliter) of PBoV G3-positive intestinal content saved in our laboratory [26] four times at 2-week intervals. The serum samples collected before first administration and after final administration were subjected to indirect immunofluorescence assay (IFA) and ELISA using the VP1 protein expressed in our laboratory as the coating antigen for evaluation [27], and designated as PBoV G3 standard negative and positive sera, respectively. The serum was stored at −20 °C until use.

HEK-293T cells were seeded in 12-well cell culture plates. When the cell density reached 80%, pcDNA3.1-PBoV-VP1 was transfected into HEK-293T cells. The samples were fixed with 4% paraformaldehyde at 36 h post transfection for 1 h at room temperature (RT), followed by treatment with TritonX-100 for 20 min at RT. After treatment with 5% BSA for 2 h at RT, the samples were treated with the prepared antiserum (1:100) to PBoV G3 overnight at 4 °C, followed by treatment with Alexa Flour488 goat anti-pig IgG antibody for 1 h at RT. Finally, the samples were treated with DAPI and subjected to a fluorescence microscope.

### 2.3. Determination of Optimal Conditions for Peptide-Based ELISA

The optimum concentrations of the peptide, serum, and secondary antibody were determined via checkerboard titration. The ELISA procedure was as follows: 96-well microtiter plates (Thermo Scientific, Waltham, MA, USA) were coated with different concentrations of peptides (25 ng/well, 50 ng/well, 100 ng/well, or 200 ng/well) and incubated at 4 °C overnight. The plates were blocked with different concentrations of bovine serum albumin (BSA) (1%, 2.5%, and 5%) in phosphate-buffered saline with 0.05% Tween-20 (*v*/*v*) (PBST) (200 μL well) at 37 °C for 2 h after three washes with PBST. Then, different dilutions (1:100, 1:200, and 1:400) of serum samples were added (100 μL/well), and the plates were incubated at 37 °C for 1 h. After three washes with PBST, diluted horseradish peroxidase (HRP)-conjugated goat anti-pig IgG antibody (Beyotime, Shanghai, China) (1:5000; 1:10,000; 1:25,000; and 1:50,000) was added (100 μL/well), and the plates were incubated at 37 °C for 1 h. After an additional three washes with PBST, the substrate 3,3′,5,5′-tetramethylbenzidine (TMB) (Beyotime) was added (50 μL/well), and the plates were incubated at room temperature in the dark for the indicated times (2.5, 5, 7.5, and 10 min), followed by the addition of 2 M H_2_SO_4_ (50 μL/well) to stop the reaction. Finally, the plates were subjected to an ELISA reader (Thermo Fisher, Spectrophotometer, VLBL00D1, Waltham, MA, USA) to determine the OD value at 450 nm.

### 2.4. Determination of the Cutoff Value

The lack of suitable cell culture systems for PBoV propagation makes it difficult to produce virus antigens for antibody detection. There are few ELISA-based techniques for detection of antibodies against PBoV, and a commercial PBoV IgG ELISA kit is not yet available. Due to the lack of a gold standard for PBoV antibody detection, the positive and negative serum samples that were used to set the cutoff value were screened via ELISA using the PBoV G3 VP1 protein as a coating antigen, which was previously expressed in our laboratory [26]. Fifty identified negative samples were randomly selected and subjected to the peptide-based ELISA optimized in this study. The OD values of the 50 samples were calculated via the formula $\bar{X}$ + 3SD. A sample was defined as positive when the OD value was >$\bar{X}$ + 3SD and negative when the OD value was ≤$\bar{X}$ + 3SD.

### 2.5. Evaluation of Specificity, Sensitivity, and Reproducibility

To evaluate the specificity of the ELISA, antisera positive for FMDV-O, FMDV-A, PRV, ASFV, SF, PCV2, PEDV, and TGEV were employed. The sensitivity of the ELISA was compared with the IFA assay via detection of 2-fold serial dilutions of standard PBoV G3-positive sera starting from a dilution of 1:50. Five PBoV G3-positive serum samples and five PBoV G3-negative serum samples were used to assess the reproducibility of the ELISA, and the coefficient of variation was calculated via the formula SD/$\bar{X}$ × 100%.

### 2.6. Detection of Field Samples

A total of 1373 porcine serum samples collected from Anhui province, Gansu province, Guangdong province, Guizhou province, Hebei province, Henan province, Hunan province, Jiangsu province, Shaanxi province, Sichuan province, Xinjiang province, and Zhejiang province in China from 2022 to 2023 were detected via the developed ELISA to investigate the positive rate of PBoV G3 and assess the applicability of the ELISA.

## 3. Results

### 3.1. The Amino Acid Sequence of the PBoV G3 VP1 Peptide

**Figure 1:**
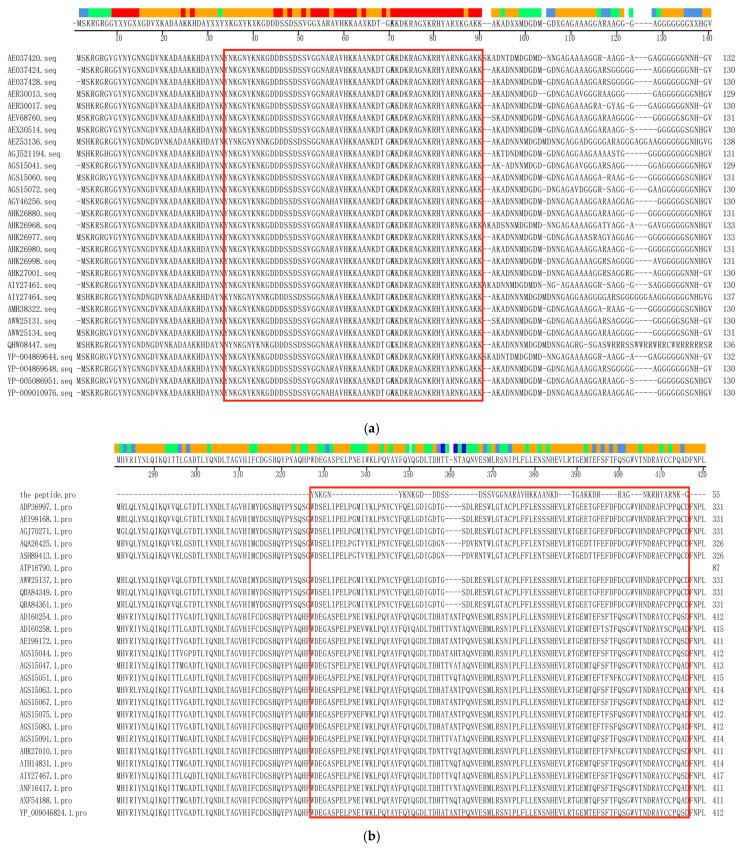
Analysis and selection of the conserved peptide of the PBoV G3 VP1 protein. (**a**) The aa sequences of the PBoV G3 VP1 protein from Genebank database were analyzed using MegAlign 6.0 to screen the conserved peptide. (**b**) To analyze the specificity of the selected peptide, MegAlign was employed to compare the similarity of the peptide and the aa sequences of the PBoV G1 and G2 VP1 protein. The peptide sequences of this study are located in the red boxes.

The amino acid (aa) sequence of the PBoV G3 VP1 protein deposited in the GenBank database was analyzed to screen the highly conserved region. After antigenicity analysis, the peptide YNKGNYKNKGDDDSSDSSVGGNARAVHKK A ANKDTGAKKDRRAGNKRHYARNKGAKK was selected for synthesis as a coating antigen (Figure 1a,b). The VP1 aa sequences of PBoV G1 and G2 from the GenBank database were employed to analyze the specificity of the conserved peptide of the PBoV G3 VP1 sequence, and the results showed that the similarity of the selected peptide with VP1 aa sequence of PBoV G1 and G2 was 10.5–26.3%, indicating that the peptide is specific and has no cross-activity with PBoV G1 and G2 (Figure 1a,b).

### 3.2. Standard Negative and Positive Porcine Sera Against PBoV G3

**Figure 2:**
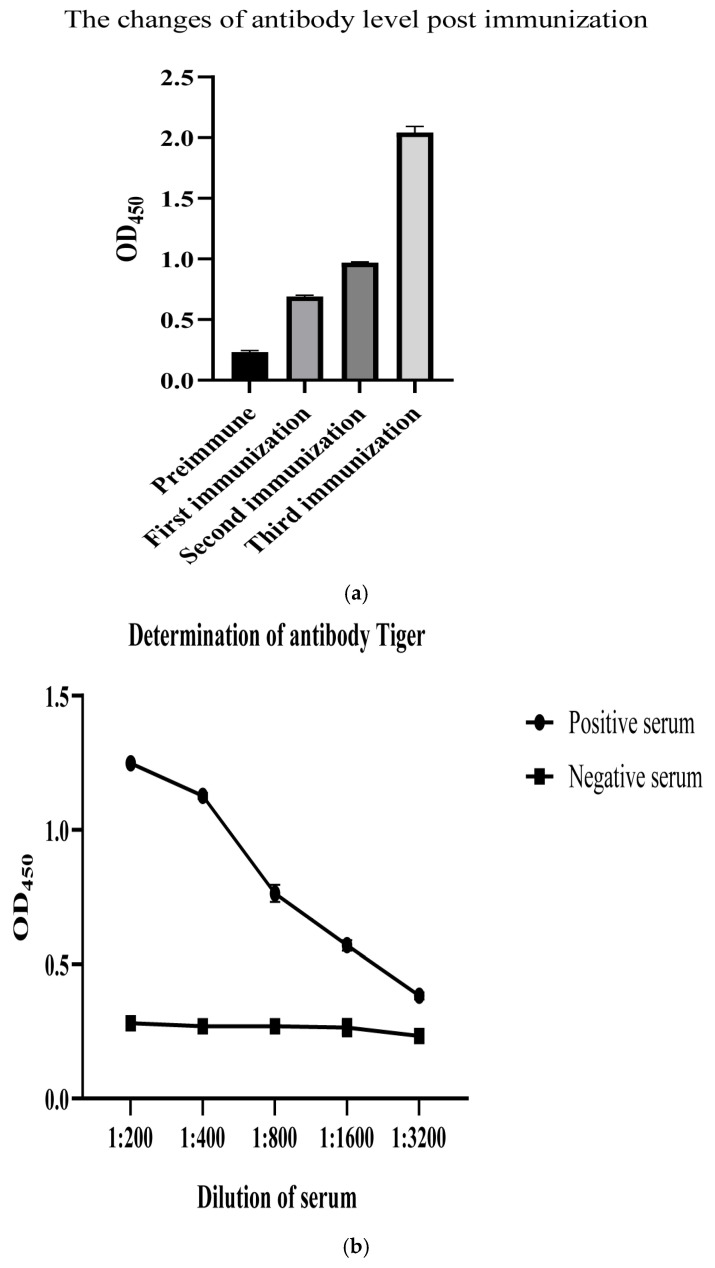

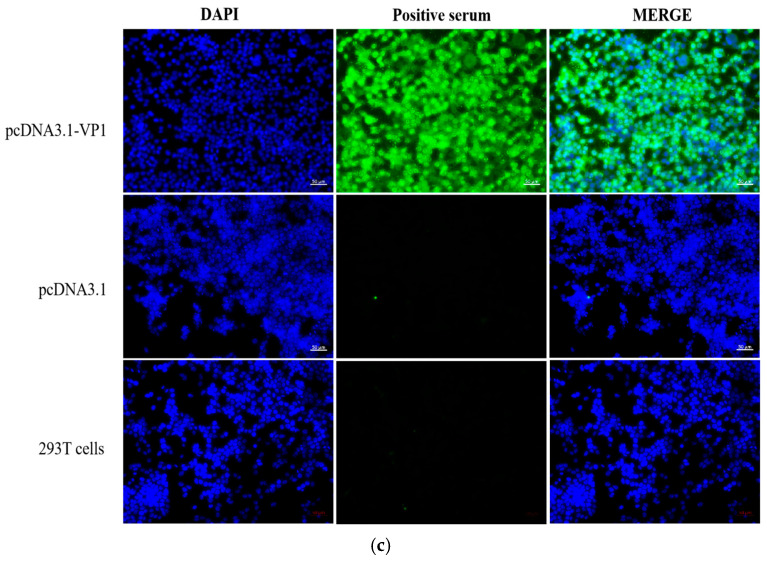
Preparation of standard positive and negative sera. To determine the antibody level to PBoV G3 post-immunization, serum samples collected before first immunization and after each immunization were detected by ELISA using the VP1 protein expressed in our lab as a coating antigen (**a**), by which the antibody titer of sera collected after final immunization was determination (**b**). IFA was employed to analyze the final obtained positive serum (**c**).

To prepare standard negative and positive porcine sera against PBoV G3, pigs were orally administered PBoV G3-positive intestinal content. IFA and ELISA were employed to analyze the sera collected before the first administration and after the final administration, and the results showed that we successfully obtained standard negative and positive porcine sera (Figure 2a–c).

### 3.3. Peptide-Based ELISA

To determine the optimal concentrations of the coating antigen, serum samples, and secondary antibody, the checkerboard titration method was employed. The results indicated that the concentrations of the peptides, serum samples, and conjugate secondary antibodies were 50 ng/well, 1:200 dilution, and 1:25,000 dilution, respectively, according to the P/N value. A total of 50 PBoV G3-negative serum samples were measured via optimal ELISA, and OD values were read and calculated. The cutoff value of the ELISA was set at 0.4329 (Table 1), which meant that the samples could be defined as positive when the OD value was >0.4239 and negative when the OD value was ≤0.4239.

### 3.4. Specificity, Sensitivity, and Reproducibility of the ELISA

**Figure 3:**
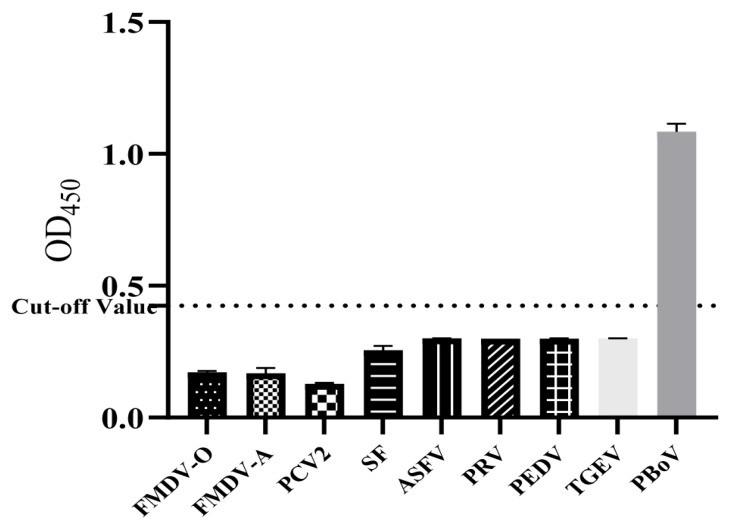
Specificity evaluation of the ELISA. For specificity evaluation of the ELISA, porcine positive sera for FMDV-O, FMDV-A, PCV2, SF, ASFV, PRV, PEDV, TGEV, and PBoV were subjected to the ELISA. The results are representative of three independent experiments. The dashed line represents the negative and positive thresholds.

To evaluate the specificity of the ELISA, FMDV-O-, FMDV-A-, PRV-, SF-, ASFV-, PCV2-, PEDV-, and TGEV-positive antisera were employed. The results revealed that there was no cross-reaction with these antisera (Figure 3), indicating that the ELISA has good specificity. The sensitivity of the ELISA was evaluated by comparison with an IFA assay, both of which were applied to detect 2-fold serial dilutions of standard porcine PBoV G3-positive serum, and the results revealed that the detection limit of the ELISA was 1:1600 and of IFA was 1:50, indicating that the ELISA has high sensitivity. The interassay and intraassay reproducibility were determined by detecting 10 randomly selected porcine serum samples, and the results revealed that the interassay and intraassay CVs were less than 10% (Table 2), indicating that ELISA has high reproducibility.

### 3.5. Application of the ELISA

**Figure 4:**
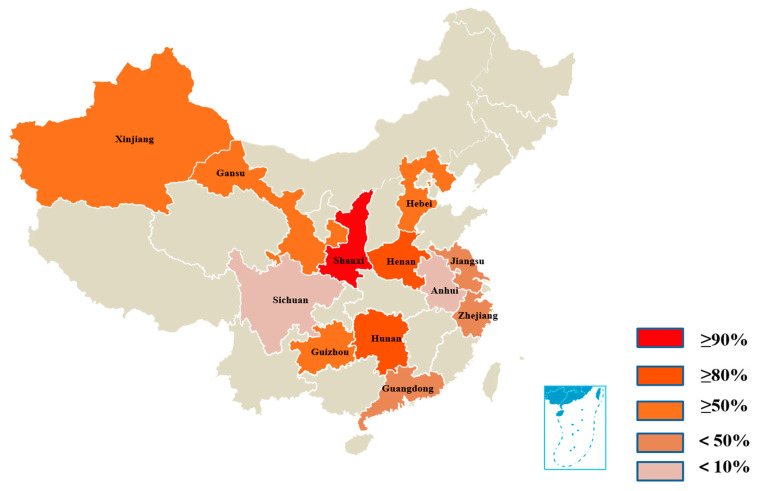
Geographic distribution of sample collection and the prevalence of PBoV G3. A total of 1373 porcine serum samples were collected from pig farms of 12 provinces in China between 2022 and 2023. And all the serum samples were subjected to the ELISA to investigate the prevalence of PBoV G3.

After evaluation of the specificity, sensitivity, and reproducibility of the ELISA, 1373 serum samples collected from 12 provinces in China were detected to investigate the prevalence of PBoV G3. The results revealed that a total of 653 (47.56%) samples were positive for PBoV G3, and the positive rates of PBoV G3 in Anhui, Gansu, Guangdong, Guizhou, Hebei, Henan, Hunan, Jiangsu, Shaanxi, Sichuan, Xinjiang, and Zhejiang were 9.48%, 46.75%, 16.67%, 54.83%, 60.00%, 83.33%, 80.33%, 24.36%, and 90.00%, respectively. Additionally, the percentage of positive samples collected from Shanxi Province was the highest (90%), whereas that from Sichuan Province was the lowest (5.69%) (Table 3, Figure 4).

## 4. Discussion

PBoV is associated with respiratory or diarrheal diseases in pigs [13,28,29]. However, the exact pathogenesis of PBoV remains unclear. Individual PBoV infections often appear asymptomatic, whereas coinfections may lead to more pronounced clinical symptoms. Because immortalized cell lines are not available, basic studies are limited. Therefore, reliable detection methods are needed to survey the prevalence of PBoV. ELISA is a powerful method for detecting pathogen-specific antibodies. Currently, several ELISA methods have been established to detect PBoV. In 2011, John M et al. reported an antigen-detecting ELISA by using monoclonal antibodies against PBoV 3 and 4 [9]. In 2016, Zhang W et al. established an ELISA for the detection of anti-PBoV IgG antibodies by using PBoV-LPs as antigens [13]. In 2019, Shi et al. developed an ELISA based on recombinant nucleoprotein 1 (NP1) to investigate the seroprevalence of PBoV in China [24]. However, owing to the genetic diversity of PBoV, ELISA methods for the detection of PBoV are not yet available. PBoV G3 is the most prevalent in China; here, we report a universal ELISA for the detection of PBoV G3.

Point mutations play a significant role in the evolution of viruses in the Parvovirinae subfamily. We analyzed the aa sequences of the VP1, VP2, NS1, and NP1 proteins encoded by PBoV G3 and found that the short region of the PBoV G3 VP1 protein at the 5′ terminus was conserved. It is difficult to develop a universal ELISA method for the detection of PBoV G3 using complete protein antigens. However, owing to the genetic diversity of PBoV, universal ELISA methods for the detection of PBoV are not yet available. The VP1 protein of PBoV G3 shared low amino acid homology with PBoV G1 and PBoV G2, indicating a low probability of cross-reactivity between the NP1 of PBoV G1 used in the present study and those of PBoV G2 or PBoV G3, although the possibility cannot be ruled out. Previous reports have suggested that peptides can be employed as antigens for ELISA [30,31,32]. Therefore, the conserved peptide of the PBoV G3 VP1 protein was used for ELISA. The evaluation data revealed that the ELISA exhibited good specificity, sensitivity, and reproducibility.

After the evaluations, 1373 serum samples collected from 12 provinces in China were tested to determine the application of the VP1 protein peptide-based ELISA and investigate the prevalence of PBoV G3. The total positive rate of 1373 serum samples was 47.56% (653/1373), and the positive rate in each province was 33.87% (21/62) in Zhejiang, 24.36% (29/119) in Jiangsu, 5.69% (7/123) in Sichuan, 60% (72/120) in Hebei, 83.335% (130/156) in Henan, 46.75% (101/216) in Gansu, 9.48% (11/116) in Anhui, 54.83% (68/124) in Guizhou, 16.67% (10/60) in Guangdong, 80.33% (98/122) in Hunan, 90% (54/60) in Shaanxi, and 54.73% (52/195) in Xinjiang (Table 3). Among them, the positive rate in Shanxi was the highest, and that in Sichuan was the lowest. Previous investigation showed that PBoV is prevalent in Zhejiang, Jiangsu, Hebei, Henan, Gansu, Anhui, Guizhou, Guangdong, and Xinjiang Provinces [6,16,17], which is consistent with our results. The prevalence of PBoV G3 was reported for the first time in Shaanxi, Sichuan, and Hunan Provinces, indicating the wide distribution of PBoV G3 in China. In addition, we observed that the positive rate of the central provinces was higher than that of the other provinces (Figure 4), which is consistent with a previous report [33]. The significant differences in the positive rates across different provinces may be associated with geographical factors [16,17].

## 5. Conclusions

In conclusion, a powerful ELISA method for the detection of antibodies against PBoV G3 was established, which facilitates investigations of the distribution and prevalence of PBoV G3. Our results revealed that PBoV G3s circulate widely in the pig population in China, and further studies are needed to comprehensively understand the prevalence of the virus.

## Figures and Tables

**Table 1:** Determination of cutoff values for the ELISA.

Negative Serum										
OD450 nm	0.2453	0.2500	0.2146	0.2644	0.2622	0.2893	0.2775	0.2639	0.2840	0.3554
	0.2686	0.2624	0.2624	0.2477	0.2893	0.2477	0.2574	0.2149	0.3185	0.3096
	0.2583	0.3139	0.2440	0.2194	0.2855	0.2260	0.2553	0.3943	0.3101	0.3066
	0.3221	0.3485	0.1981	0.1895	0.2343	0.1998	0.3279	0.3200	0.2996	0.2934
	0.3237	0.3113	0.3551	0.3399	0.3386	0.3367	0.3396	0.3486	0.2541	0.2967
Mean value ($\bar{X}$)	0.2835									
Standard deviation (SD)	0.0468									

$\bar{X}$ is the mean value; SD stands for variance.

**Table 2:** Determination of the reproducibility of the ELISA.

Samples	OD450 nm						Mean	SD	CV (%)	CV Mean (%)
Intra batch										
1	1.1547	1.1519	1.2668	1.2671	1.2501	1.2497	1.2234	0.0548	4.4816	4.3100
2	1.0050	1.0072	1.0571	1.0577	1.1272	1.1264	1.0634	0.0542	5.0954	
3	1.1150	1.1178	1.1210	1.1216	1.1671	1.1663	1.1348	0.0248	2.1876	
4	1.0312	1.0326	1.1377	1.1383	1.1246	1.1242	1.0981	0.0516	4.7027	
5	1.1609	1.1625	1.3719	1.3723	1.3312	1.3316	1.2884	0.0998	7.7473	
6	0.2122	0.2138	0.2122	0.2130	0.2276	0.2278	0.2178	0.0077	3.5439	
7	0.2360	0.2368	0.2097	0.2105	0.2098	0.2104	0.2189	0.0136	6.2080	
8	0.2520	0.2528	0.2596	0.2600	0.2700	0.2708	0.2609	0.0081	3.1054	
9	0.2232	0.2240	0.2123	0.2129	0.2281	0.2285	0.2215	0.0072	3.2575	
10	0.2070	0.2072	0.2044	0.2046	0.2172	0.2164	0.2095	0.0058	2.7710	
Inter batch										
1	1.2069	1.2071	1.2001	1.2005	1.1960	1.1964	1.2012	0.0049	0.4063	3.4221
2	1.5518	1.5522	1.5747	1.5753	1.5201	1.5207	1.5491	0.0245	1.5828	
3	1.2197	1.2203	1.2257	1.2261	1.2771	1.2773	1.2410	0.0281	2.2674	
4	1.3521	1.3525	1.3599	1.3605	1.3311	1.3313	1.3479	0.0134	0.9950	
5	1.3428	1.3432	1.3743	1.3747	1.3237	1.3241	1.3471	0.0229	1.6966	
6	0.1717	0.1723	0.1937	0.1943	0.1810	0.1814	0.1824	0.0099	5.4205	
7	0.1991	0.1997	0.1798	0.1804	0.1715	0.1717	0.1837	0.0127	6.9379	
8	0.1772	0.1776	0.1989	0.1993	0.1931	0.1935	0.1899	0.0101	5.2920	
9	0.1957	0.1963	0.1988	0.1996	0.1812	0.1816	0.1922	0.0085	4.4194	
10	0.2279	0.2283	0.2549	0.2555	0.2489	0.2491	0.2441	0.0127	5.2037	

Abbreviations: CV, coefficient of variation.

**Table 3:** Detection in field serum samples.

Provinces	Number of Serum Samples	Number of Positive Samples	Positive Rate (%)
Anhui	116	11	9.48
Gansu	216	101	46.75
Guangdong	60	10	16.67
Guizhou	124	68	54.83
Hebei	120	72	60.00
Henan	156	130	83.33
Hunan	122	98	80.33
Jiangsu	119	29	24.36
Shaanxi	60	54	90.00
Sichuan	123	7	5.69
Xinjiang	95	52	54.73
Zhejiang	62	21	33.87
Total	1373	653	46.67

## Data Availability

The raw data supporting the conclusions of this article will be made available by the authors upon request.
